# Antidiabetic Potential of *Senna siamea*: *α*-Glucosidase Inhibition, Postprandial Blood Glucose Reduction, Toxicity Evaluation, and Molecular Docking

**DOI:** 10.1155/sci5/6650349

**Published:** 2025-01-23

**Authors:** Suthinee Sangkanu, Armad Heemman, Sathianpong Phoopha, Thanet Pitakbut, Wandee Udomuksorn, Sukanya Dej-adisai

**Affiliations:** ^1^Department of Pharmacognosy and Pharmaceutical Botany, Faculty of Pharmaceutical Sciences, Prince of Songkla University, Hat Yai, Songkhla 90112, Thailand; ^2^Traditional Thai Medical Research and Innovation Center, Faculty of Traditional Thai Medicine, Prince of Songkla University, Hat Yai, Songkhla 90112, Thailand; ^3^Pharmaceutical Biology, Department of Biology, Friedrich-Alexander-Universität Erlangen-Nürnberg (FAU), Erlangen 91058, Germany; ^4^Pharmacology Program, Division of Health and Applied Science, Faculty of Science, Prince of Songkla University, Hat Yai, Songkhla 90112, Thailand

## Abstract

*Senna siamea* (Lam.) H.S. Irwin & Barneby is used in Thai cuisine. This plant is also used in traditional treatments, including diabetes. Therefore, this study aims to examine the antihyperglycemic effects of *S. siamea* heartwood extract. The ethanolic extract of *S. siamea* heartwood exhibited activity against *α*-glucosidase enzyme with IC_50_ values of 54.4 μg/mL. Moreover, *S. siamea* extract (250–1000 mg/kg BW) was tested using normal rats with and without sucrose of 3 g/kg BW administration. The results showed that all extract concentrations significantly reduced fasting blood glucose compared with the control. In addition, results also agreed with the amount of sucrose in the small intestine of rats. In the acute toxicity study, a single dose of the *S. siamea* extract at 2000 mg/kg BW caused no mortality, and hematological and biochemical parameters also revealed no toxic effects of the extract on rats. The subchronic toxicity study, administration of the extract for 90 days, at 250 mg/kg BW, caused no significant changes in the hematological and biochemical parameters of rats in the treated groups compared with the control group. However, histopathology of the liver and kidney indicated an inflammatory response at 500 and 1000 mg/kg BW of the extract, correlating to hematological and biochemical findings. Finally, molecular docking was conducted to evaluate theoretical interactions between three main stilbenes previously found in *S. siamea* extract and mammalian *α*-glucosidases (Wistar rat and human). The simulation supported the in vivo study and suggested the potential for human glucosidase inhibition. Therefore, *S. siamea* could be a promising candidate against *α*-glucosidase. This study offers encouraging information on the potential of natural compounds from *S. siamea* to act as *α*-glucosidase inhibitors for diabetes treatment through drug development or dietary supplement for hyperglycemia individuals.

## 1. Introduction

Diabetes is a disease characterized by hyperglycemia resulting from defects in insulin secretion, insulin resistance, or both. Diabetes's chronic hyperglycemia is linked to chronic damage, malfunction, and organ failure, particularly to the kidneys, eyes, heart, nerves, and blood vessels [1]. The disease has three major types such as type 1 diabetes mellitus (T1DM), type 2 diabetes mellitus (T2DM), and gestational diabetes mellitus (GDM). T2DM is the most prevalent type of DM, which accounts for almost 90% of DM [2]. Drugs known as *α*-glucosidase inhibitors (AGIs) can be used to treat patients who have T2DM or impaired glucose tolerance. Acarbose is the most widely prescribed AGI and other AGIs are miglitol, voglibose, and emiglitate [3]. AGIs work by restricting several enzymes that the intestines need to break down carbohydrates into simpler forms. It inhibits membrane-bound *α*-glucosidases, including intestinal glucoamylase, sucrase, maltase, and isomaltase, as well as pancreatic alpha-amylase, which converts complex starches and oligo-, tri-, and disaccharides into simple sugars that are readily absorbed. Because AGIs inhibit the action of these enzymes, they reduce the absorption of dietary carbohydrates and the ensuing rise in blood glucose and insulin levels. However, AGIs have been reported side effects such as diarrhea, abdominal pain, and flatulence [4]. The use of natural substances as medicinal agents is becoming more popular for the treatment and prevention of T2DM. Extracts from two plants, *Chrysophyllum cainito* and *Ensete superbum*, showed the notable inhibitory effects with low IC_50_ values of 0.0012 and 0.0018 mg/mL, respectively, compared with IC_50_ values of acarbose (0.198 and 0.1215 mg/mL) [5, 6]. *Chrysophyllum cainito* leaves extract was safe at dose of 75 mg/kg BW in rat model. It reduced sugar blood levels in diabetic rats but did not affect weight [7]. Similarly, *Ensete superbum* seed extract had no signs of acute toxicity at doses of 2000 mg/kg BW [8].

*Senna siamea* (Lam.) H.S. Irwin & Barneby is a member of Fabaceae family, subfamily, Caesalpinioideae. The tree is also known as *Cassia siamea*, *Cassia florida*, and *Senna sumatrana*. It is native to South and Southeast Asia [9]. *S. siamea* is commonly used as medicinal herb in Thailand. Flowers, young fruits, and leaves of *S. siamea* are commonly consumed in Thai cuisine as a Khi Lek curry but the plant parts must be boiled with water at least 2–3 times before cooking [10]. *S. siamea* leaves provide nutritional values with a high percentage of carbohydrate (88.50%), followed by fiber content (10.0%), moisture content (11.50%), ash content (5.00%), protein content (8.66%), and fat or lipid content (1.0%), respectively [11]. In addition, *S. siamea* is also a good source of minerals such as iron, magnesium, manganese, potassium, calcium, sodium, copper, and phosphorus, which are essential for body tonics [12]. People most commonly use the plant's leaves to make herbal treatments, especially in Asia and Africa. Dried leaves boiled in water are drunk with lemon juice to treat coughs, stomach pains, malaria, and liver disorders. In Thailand, dried leaves are used as a laxative and as a sleeping pill in capsule form. In Southeast Asia, the root or stem bark is boiled and drunk for the treatment of diabetes mellitus [13]. *S. siamea* has been reported of chemical constituents as cassiarins A which was isolated from the leaf and emodin plus lupeol which were isolated from the stem bark, all exhibited the potent antiplasmodial activity [14, 15]. The stem bark extract contains triterpenes, flavonoids, anthraquinones, and phytosterols and presented the anti-inflammatory and analgesic effects [16] Ethanolic, ethyl acetate, and hexane extracts from leaves exhibited antibacterial activity against Gram-positive and Gram-negative bacteria [17]. Interestingly, a previous report has been showed that methanolic leaves extract had antidiabetic and antilipemic effects in rats [18]. In a recent study, we have shown that phytochemical compounds isolated from *S. siamea* heartwood such as resveratrol, piceatannol, dihydropiceatannol, chrysophanol, and emodin had anti-insulin resistance effects on zebrafish larvae. According to a molecular docking research, chrysophanol and emodin decreased PTP1B activity, demonstrating sustained binding associations with an allosteric region of the PTP1B enzyme [19]. This enzyme is a significant inhibitor of insulin and leptin signaling. It seems that lowering PTP1B activity prevents fat and diabetes while simultaneously increasing the effect of insulin and leptin [20]. Resveratrol inhibited DPP-IV using the enzymatic test and molecular docking. It exhibited a binding relationship with DPP-IV's catalytic pocket, the GLU205 residue [19]. Dipeptidyl peptidase IV (DPP-IV) improves plasma glucose metabolism by stimulating insulin secretion from pancreatic beta cells and suppressing glucagon release in response to the breakdown of glucagon-like peptide 1 (GLP-1) and glucose-dependent insulinotropic polypeptide [21]. As a result, PTP1B and DPP-IV enzymes are therapeutic targets for the management of T2DM.

Therefore, this study provides the ability to reduce blood sugar levels and to evaluate their toxicity in rat model of *S. siamea* heartwood extract. Finally, the binding interactions of reported pure compounds of *S. siamea* heartwood extract were analyzed using molecular docking studies to estimate the binding potential of the pure compounds to Wistar rat and human *α*-glucosidase enzyme proteins.

## 2. Materials and Methods

### 2.1. Plant Extract

*S. siamea* heartwoods were purchased from the Saiburi Thai herbal shop in Songkhla, Thailand (authorized to access by the Plant Varieties Protection Office, Thailand). This plant sample was identified by specialists from Department of Pharmacognosy and Pharmaceutical Botany, Faculty of Pharmaceutical Sciences, Prince of Songkla University and herbarium specimen (Herbarium no. SKP072191901) was deposited at the same place [19]. The extract was obtained from 10 kg of dried powder heartwood soaked in ethanol for 3 days. The extract was filtered through filter paper and evaporated to dryness in a rotary vacuum evaporator (× 3). The plant extract was stored at 4°C until examined.

### 2.2. *α*-Glucosidase Inhibition

Plant extracts were evaluated for *α*-glucosidase inhibitory activity according to the method given by Dej-adisai and Pitakbut [22]. In brief, plant extracts (50 μL) at 2 mg/mL were incubated with 50 μL of the *α*-glucosidase 1 unit from *Saccharomyces cerevisiae* (Type I, lyophilized powder, Sigma, EC 3.2.1.20) for 2 min at 37°C with an additional 50 μL of 0.1 M phosphate buffer (pH 7.0). After that, the reaction was started with the addition of 50 μL of p-nitrophenyl-*α*-D-glucopyranoside (pNPG) (substrate), the product (pNP) was performed and monitored at 405 nm every 30 s for 10 min by microplate reader (VARI-OSKAN LUX, Thermo Scientific, USA). The measured absorbance of tested wells was deleted with the measured absorbance of blank before using for the velocity calculation (equation (1) The initial velocity (V) from the initial reaction of each sample was determined and the percentage of inhibition was further calculated by equation (2) The IC_50_ value was obtained from the calibration plotted between percentages of inhibition and seven concentrations of samples (seven concentrations: 15.625–1000 μg/mL). All experiments were performed in triplicate.

(1) $$\begin{array}{c} \text{Velocity}=\frac{\Delta \text{Absorbance at }405\text{ nm}}{\Delta \text{Time}}, \end{array}$$

(2) $$\begin{array}{c} \%\text{Inhibition}=\left[\frac{\left(V_{\text{control}}-V_{\text{sample}}\right)}{V_{\text{control}}}\right]\times 100. \end{array}$$

### 2.3. Animals Model

Healthy adult male and female albino Wistar rats (180–200 g) from the Southern Laboratory Animal Facility, Faculty of Science, Prince of Songkla University, were used for the in vivo experiments. Animals were kept in cages under similar conditions in animal houses and provided with a standard diet and water ad libitum. Each cage contained 10 rats of the same sex with a bedding of husk, and 12 h light/dark cycles were provided. The temperature of the animal house was maintained at 25°C ± 2°C and a relative humidity was set to 60% ± 10%. The rats were housed in this condition for 1 week before testing.

### 2.4. Oral Sucrose Tolerance Test

After 1 week of adaptation period, male rats were divided into five groups (*n* = 10) and fasted for 12 h. The blood samples from the tail vein of all groups of rats were collected and measured the blood glucose level by using a glucose meter (Accu-Chek Roche, USA) (*t* = 0 min). Then, *S. siamea* extract at doses of 250, 500, and 1000 mg/kg BW and acarbose at 50 mg/kg BW were orally administered. Sucrose solution (3 g/kg BW) was orally administered 30 min after extract and acarbose administration, and blood was taken and measured the glucose level at 30, 60, 90, 120, and 180 min afterward [23].

After the previous experiment, rats were sacrificed and the small intestinal was removed. Small intestinal segments were twice enema with distilled water. Then, the water obtained from the enema was centrifuged at 4°C, 3000 rpm for 15 min (Sorvall RC-3C Plus High-Capacity Centrifuge, GMI, USA). The supernatant was collected and measured the sucrose content with a sucrose assay kit (BioVision, USA).

### 2.5. Acute Toxicity and Subchronic Toxicity

The acute oral toxicity test was carried out according to the Organization for Economic Co-operation and Development (OECD) guidelines 425 [24]. Female rats were divided into two groups (*n* = 5). The first group was a control group that served distilled water. The second group received 2000 mg/kg BW of *S. siamea* heartwood extract. The test was administered orally to rats and then the rats were observed for toxic effect for the first 30 min followed by hourly for 4 h for the first 24 h. If there are no signs of toxic effect or mortality observed on the rat within 24 h., they were observed daily for 14 days. The blood was collected for biochemical and hematological determination.

The subchronic toxicity study was carried out according to OECD test guideline 408 (90 day study) [25]. A total of 80 rats (40 males and 40 females) were divided into four groups, one control group and three treatment groups, with each group containing 20 rats (10 males and 10 females). Control group received the vehicle 20% PEG-400. Treatment groups were administered orally with *S. siamea* heartwood extract daily, at doses of 250, 500, and 1000 mg/kg BW. The behavior and weights of the rats were recorded daily. At the end of experiment (90 days), all the rats were anesthetized and their blood samples were collected.

### 2.6. Histopathological Examination

The animal organs such as liver and kidneys were excised immediately after sacrificed, cleaned in saline, and fixed in 10% formalin. The histological sections were produced by Department of Anatomy, Faculty of Science, Prince of Songkla University. The tissues were examined under a microscope in a random order animal in each group.

### 2.7. Protein Alignment and Molecular Docking Simulation

Wistar rat glucosidase (sucrase–isomaltase, AFDB accession no: AF-P23739-F1) was obtained from the AlphaFold database [26, 27], while human glucosidase (sucrase–isomaltase, PDB ID: 3LPP) was downloaded from the RCSB Potein Data Bank. The Chimera program (Version 1.17.3) was used to prepare proper protein files for molecular docking and perform 3D and 2D protein alignments [28]. On the other hand, the chemical structures of resveratrol (CID: 445154), piceatannol (CID: 667639), and dihydropiceatannol (CID: 274130318) were received from the PubChem database. Unlike protein, Open Babel software (Version 3.1.0) was used to prepare all ligand files and the 3D structure was generated using a general AMBER force field (GAFF).

The latest version (1.2.5) of AutoDock Vina was used for docking simulation [29]. The binding pocket and size were defined as *x*, *y*, and *z* coordination (*x* = 39.8, *y* = 58.5, and *z* = 78.8), following our previous report [30], and a size of 25 × 25 × 25. Docking parameters were set as 3 for energy range, 8 for exhaustiveness, and 10 for the number of docking poses. Finally, a 2D interaction diagram was produced by the Ligplot^+^ program (Version 2.2.8) [31].

### 2.8. Statistics

The data were evaluated using one-way variance analysis (ANOVA) for mean differences between different extracts, followed by a *T*-test. The results were expressed as the mean ± standard deviation of three determinations. In all analyzes, ⁣^∗^*p* < 0.05 and ⁣^∗∗^*p* < 0.01 were considered statistically significant.

## 3. Results

### 3.1. In Vitro Study: *α*-Glucosidase Inhibitory Effect

The ethanolic extract of *S. siamea* heartwoods was evaluated for their *α*-glucosidase inhibitory activity. The extract exhibited inhibitory activity with 98.82% ± 0.69% inhibition (IC_50_ 54.4 μg/mL), better than the activity shown by acarbose (positive control) with 87.55% ± 2.58% inhibition (IC_50_ 231.5 μg/mL) at the same concentration as 2 mg/mL.

### 3.2. In Vivo Study: Oral Sucrose Tolerance in Normal Rat

**Figure 1:**
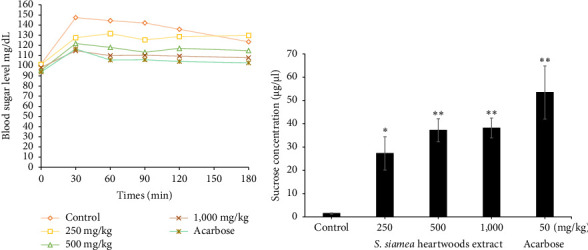
Effect of the administration with the *S. siamea* heartwood extract on fasting blood glucose (FBG) in normal rats (*n* = 10 rats). (a) Postprandial glucose responded to extract in rats. (b) Sucrose level in the small intestine after treatment with extract and acarbose. ⁣^∗^*p* < 0.01 and ⁣^∗∗^*p* < 0.05 compared with the control.

The effects of the extract on blood glucose level in normal rats are shown in Figure 1(a). In particular, the rats given the extract at 250, 500, and 1000 mg/kg BW dose reduced blood sugar significantly as observed at 30–180 min when compared with normal control rats. Similarly, acarbose-treated rats also showed significant blood glucose–lowering efficacy at all the time intervals. For indicating that the hypoglycemic effect linked to the inhibition of sucrose digestion, we observed the sucrose level in the small intestine after administration extract and acarbose for 180 min. It was found that the sucrose level was higher in rats which administrated with acarbose followed by 1,000, 500, and 250 mg/kg BW of the extract (Figure 1(b)).

### 3.3. In Silico Study: Molecular Docking of Bioactive Metabolites From the *S. siamea* Heartwood Extract

**Figure 2:**
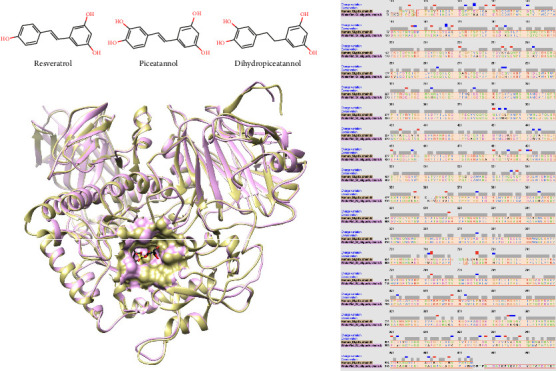
The left upper shows the chemical structures of three main stilbenes found in the *S. siamea* heartwood extract, and the left lower presents the 3D structural alignment of human (brown) and Wistar rat (pink) glucosidase. The right side demonstrates the 2D sequence alignment of human and Wistar rat glucosidase.

The authors selected three major stilbenes, such as resveratrol, piceatannol, and dihydropiceatannol (Figure 2), found in *S. siamea* heartwood, based on the authors' previous report [19] to perform a molecular docking simulation. The molecular docking here aimed to theoretically evaluate the interaction between the main metabolites in *S. siamea* heartwood and Wistar rat intestinal glucosidase (sucrase–isomaltase), supporting the authors' in vivo study and projecting the potential interaction in human intestinal glucosidase (sucrase–isomaltase). Unlike human glucosidase, PDB ID: 3LPP [32], Wistar rat glucosidase had no crystal structure available. Therefore, the authors obtained a protein model structure from the AlphaFold database, AFDB accession no: AF-P23739-F1 [26, 27] and used an experimental human glucosidase crystal structure [32] as a navigator.

The authors first performed a 3D structural alignment as shown in Figure 2. The *Q*-score (quality score) of human and Wistar rat glucosidases was 0.945, when 1 indicated identical, and the structural distance measurement (SDM) value was 10.07, with a small value referring to high similarity. Furthermore, the percent identity of both glucosidases was 73.16% based on 2D alignment. Therefore, 3D and 2D alignments suggested a highly similar protein structure between human and Wistar rat glucosidases. All scores were obtained from the Chimera program [28].

**Figure 3:**
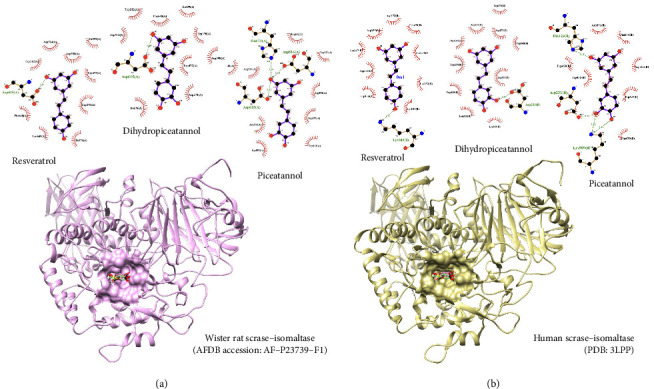
For Wistar rat glucosidase, the 3D molecular docking result of resveratrol, piceatannol, and dihydropiceatannol on Wistar rat glucosidase is shown on the lower left, and 2D interaction is presented on the upper left. 3D and 2D interactions between human glucosidase and resveratrol, piceatannol, and dihydropiceatannol are exhibited on the right upper and lower. Kotalanol in black color serves as a competitive inhibitor navigating an active site on both glucosidases. Resveratrol, piceatannol, and dihydropiceatannol are presented in red, green, and pink, respectively.

For molecular docking, the authors' protocols were validated and satisfied an accepted standard (root mean square deviation or RMSD less than 3 Å) before a theoretical evaluation. The information regarding the validation was provided in Figures S1 and S2. The molecular docking simulation set the catalytic pocket as a target for resveratrol, piceatannol, and dihydropiceatannol. As demonstrated in Figure 3, molecular docking results showed that all three stilbenes inserted themselves into the catalytic pocket of both human and Wistar rat glucosidases. Only the crucial interaction, like the hydrogen bond, was mentioned here. For resveratrol, one theoretical hydrogen bond was spotted on each glucosidase. For human glucosidase, resveratrol interacted with residue Lys 509 from the active site +1 subunit [32]. For Wistar rat glucosidase, it interacted with Asp 615. Since the authors used a predictive model for Wistar rat glucosidase, the author used the human experimental enzyme residues to guide the rat model. Therefore, residue Asp 615 on Wistar rat glucosidase is equivalent to Asp 571 on human glucosidase, located in the active site −1 subunit [32]. Similarly, dihydropiceatannol also exhibited one theoretical hydrogen bond on both enzymes, residue Asp 231 on human glucosidase and residue Asp 615 on Wistar rat glucosidase (equivalent to residue Asp 571 on human glucosidase). Both residues are located at the active site −1 subunit. Finally, only piceatannol was able to form multiple molecular interactions theoretically. Three interactions were observed from each glucosidase. For human glucosidase, piceatannol interacted with three residues: Asp231 from the −1 subunit, Lys 509 from the +1 subunit, and His 629 from the −1 subunit [32]. For Wistar rat glucosidase, three other interactions from piceatannol were spotted, such as Asp 615, Asp 472, and His 629. These residues' human glucosidase counterparts are Asp 571, Asp 472, and His629, and they are all located in the −1 subunit [32].

### 3.4. Effect of the *S. siamea* Heartwood Extract on Acute Toxicity Studies

**Figure 4:**
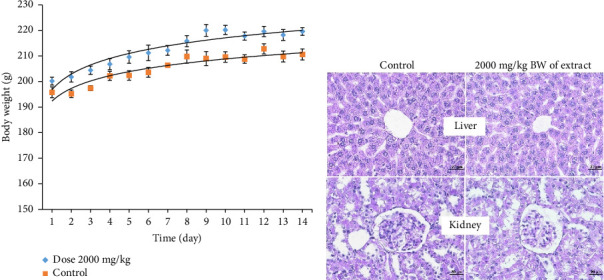
Effect of the *S. siamea* heartwood extract on acute toxicity. (a) The body weight of control and treated rats was measured at the end of the Day 14. Values are expressed as the mean ± SE (*n* = 5). (b) A photomicrograph of sections in rat liver and kidney of the control (*n* = 2) and tested groups (*n* = 2).

Following acute dose administration of 2000 mg/kg BW showed slightly increases in weights of the days when compared with Day 0. However, these changes were not significantly different in the test dose when compared with the control (Figure 4(a)). Other physical and behavior observation indicated that they showed normal characteristics such as fur and skin, eyes, salivation, respiration, urination somatomotor activity, mucous membrane, convulsion and tremors, and itching when compared with their respective control groups (Table S1). The significant changes following single administration when compared with control were found in fasting blood glucose levels (FBS) (116.60 ± 2.56 mg/dL). Serum biochemical (Table 1), hematological (Table 2), and histopathological (Figure 4(b)) studies were measured at the end of the 14 days also indicated that at dose of 2000 mg/kg of *S. siamea* heartwood extract did not cause any evident toxic signs in of the test rats.

### 3.5. Effect of the *S. siamea* Heartwood Extract on Subchronic Toxicity Studies

#### 3.5.1. Body Weight and Food Consumption

**Figure 5:**
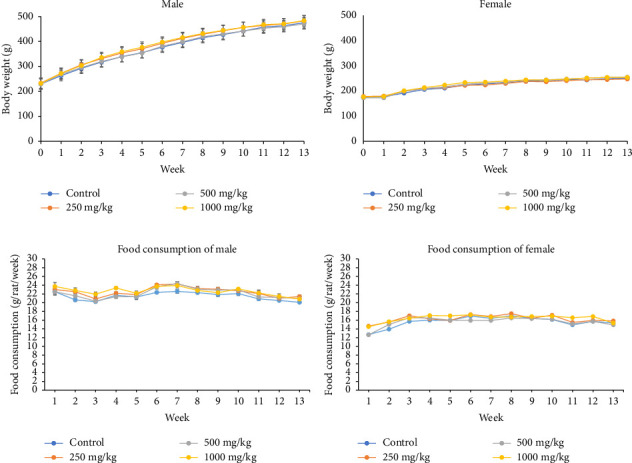
Body weight and food consumption. (a) Mean body weight of male rats; (b) mean body weight of female rats; (c) mean daily food consumption of male rats; and (d) mean daily food consumption of female rats. Values are expressed as the mean ± SE (*n* = 20).

The oral subchronic toxicity was conducted on the normal rats by administering the *S. siamea* extract once daily for 90 days. Physical and behavior observation indicated that they showed normal characteristics (Table S2). The male and female rats did not show a significant change in body weight compared to controls. However, the differential effects were found that male rats exhibited an increasing of food intake and the rate of weight gained was higher than female (Figure 5).

#### 3.5.2. Biochemical Indices

The effects of administration of the extracts at 90 days in plasma biochemical parameters in experimental male rats are shown in Table 3. Blood urea nitrogen (BUN), creatinine (Cr), serum glutamic oxaloacetic transaminase (SGOT), Na^+^, and K^+^ are significantly increased (*p* < 0.01) in rats treated with 500 and 1000 mg/kg BW of the extract. Albumin increased in the group of 1000 mg/kg BW. The treatment group of 1000 mg/kg BW showed a significantly low value for serum glutamate pyruvate transaminase (SGPT) (*p* < 0.01). Treated groups of 250, 500, and 1000 mg/kg BW showed significant decrease (*p* < 0.01) of alkaline phosphatase (ALP). In female rats, it was found that the ALP decreased in the treatment group of extract at 1000 mg/kg BW (Table 3).

#### 3.5.3. Hematological Indices

The results of hematological parameters of male and female rats on subchronic toxicity are listed in Table 4. Male rats showed no significant changes in this study at all the doses of extract when compared with those of controls. However, in female rats, an increase in mean corpuscular hemoglobin concentration (MCHC) and monocyte were observed in the treated groups of 500 and 1000 mg/kg of the extract. In addition, significant decrease was observed in the mean hematocrit and mean cell volume of female rats treated with 500 and 1000 mg/kg BW as compared with the control group.

#### 3.5.4. Histopathological Examination

**Figure 6:**
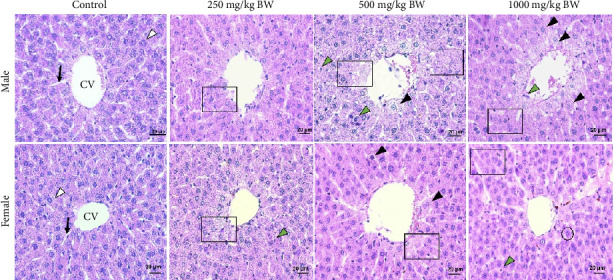
A photomicrograph of sections in rat liver of all studied groups. Control group showing normal hepatocytes (white arrowhead) with acidophilic stippled cytoplasm and vesicular nuclei radiating from central vein (CV) and separated by sinusoids (black arrowhead). The *S. siamea* heartwood extract-treated group showed nuclear and cytoplasmic condensation, shrinkage, and unclear hepatocyte boundaries (square), showing many hepatocytes with vacuolated cytoplasm (green arrowhead), indicating hydropic degeneration. Cell swelling evident (black arrowhead) is initial of necrosis of hepatocytes; others are binucleated (circle). H&E 400x.

The liver changes of male and female rats were observed in 250–1000 mg/kg of *S. siamea* heartwood extract. Male rats treated with 250, 500, and 1000 mg/kg BW of the extract showed a difference vs. the control group with scores of 0.75, 2.50, and 3.06, respectively. Meanwhile, female rats revealed scores 1, 2, and 2, respectively (Table 5). Histomorphological features of the liver changes induced by the extract are shown in Figure 6. No histological abnormality was observed in the control group (score = 0). The extract exposure led to changes in histology of liver tissues, including degenerated hepatocytes, unclear hepatocyte boundaries, necrotic cells with nuclear dissolution, binucleated cells, as well as dilated sinusoid (Figure 6).

**Figure 7:**
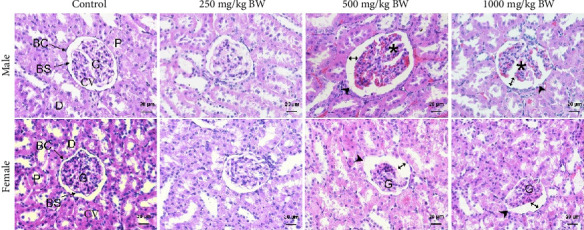
A photomicrograph of kidney sections of rats. Control male and female rats showing normal glomeruli (G), Bowman's capsule (BC), Bowman's space (BS), proximal convoluted tubule (P), and distal convoluted tubule (D). The *S. siamea* heartwood extract treated with 250 mg/kg groups showed that the kidney tissue seem to be normal. The male rats treated with 500 and 1000 mg/kg revealed atrophy of the glomerulus (⁣^∗^), large Bowman's space (↔), and damaged parietal layer of Bowman's capsule (head arrow).

In the kidney examination, the section of the control groups showed normal characteristics with a score of 0. In the extract, treated groups revealed scores 0, 3.69, and 4.06 in male and 0.38, 4.31, and 5.19 in female rats (Table 6). The normal histological structure of the glomeruli (G), Bowman's capsule (BC), Bowman's space (BS), proximal convoluted tubule (P), and distal convoluted tubule (D) was evident upon histopathological analysis of kidney samples obtained from normal control rats. In addition, kidney tissue appeared to be normal in rats treated with 250 mg/kg BW of the *S. siamea* extract. The male rats treated with 500 and 1000 mg/kg of *S. siamea* extract showed signs of damage to the parietal layer of Bowman's capsule, atrophy of the glomerulus, and a large Bowman's space. Contracted glomerulus was observed in kidney sections of female rats treated at 500 and 1000 mg/kg of the *S. siamea* extract (Figure 7).

## 4. Discussion

The ability of plants to synthesis a large variety of chemical substances has been an important cause of concern for the pharmaceutical industry. These phytochemicals are derived from different plant materials including pulps, leaves, barks, seeds, seed coats, flowers, and roots [33]. This study indicated that the *S. siamea* heartwood extract has the potential of *α*-glucosidase inhibition in vitro with IC_50_ lower than acarbose. Traditionally, this plant has a long history used to treat different diseases, including jaundice, abdominal pain, menstrual pain, and diabetes by acting as a hypoglycemic agent [34]. Result of isolation and structure elucidation of chemical constituents conducted in the previous study indicated that the *S. siamea* heartwood extract presented of stilbenoid compounds such as piceatannol, dihydropiceatannol, and resveratrol. Two anthraquinone compounds including chrysophanol and emodin have also been found [19]. Five isolated compounds showed anti-insulin resistance effects using the zebrafish model [19]. Interestingly, a molecular docking demonstrated that *α*-glucosidase activity was shown to be inhibited by resveratrol, piceatannol, and dihydropiceatannol. PTP1B activity was decreased by chyrsophanol and emodin, whereas resveratrol demonstrated the suppression of DPP-IV [19]. One of the most major problems with diabetes mellitus is the effective strategy to manage postprandial blood glucose because high blood glucose levels can promote hyperinsulinemia, increase protein glycation, and/or stimulate the development of diabetic complications by activating the polyol pathway and finally alters gene expressions. Prolonged hyperglycemic diabetes is associated with long-term damage to the pancreatic *β*-cell and inducing insulin resistance [35]. The intestinal breakdown of carbs and/or sucrose by amylases or glycoside hydrolases is a major route for the generation of glucose from meal consumption. Therefore, an idealistic and successful treatment for type 2 diabetes patients would involve preventing an excessive postprandial blood glucose rise or keeping the blood glucose limit within the normal range by regulating glucose production from disaccharides using an oral *α*-glucosidase inhibitor [36]. In this study, the Wistar rat models have been used to prove their biological and toxicity research of the *S. siamea* heartwood extract. Wistar rats tend to eat a higher percentage of high-fat foods than other strain such as Sprague–Dawley rats, which makes them more susceptible to the development of obesity through diet [37]. The results showed that the extract was effective as a blood sugar-lowering agent when administered orally in 250, 500, and 1000 mg/kg doses. Our results agreed with earlier studies where blood sugar-lowering effects of extracts from leaves [18] or root [38] of this plant have been reported. *S. siamea* heartwood extract produced effects similar to that of standard acarbose. This drug postpones the breakdown of carbohydrates by competitively blocking *α*-glucosidase in the small intestine, including glucoamylase, sucrase, maltase, and isomaltase [39]. This result explains why the oral-administration rats with the extract and acarbose had higher sucrose levels in the small intestine than the rats that did not receive the extract.

The Wistar rat and human glucosidase proteins have a highly similar structure when examined with the Chimera program [28]. The molecular docking evaluation of reported compounds of *S. siamea* heartwood (piceatannol, dihydropiceatannol, and resveratrol) against Wistar rat and human glucosidase revealed the possible interactions between the enzymes and the compounds; like a reference, a competitive inhibitor came with a human glucosidase crystal structure [32]. Furthermore, resveratrol, piceatannol, and dihydropiceatannol formed multiple hydrophobic interactions and rescued at least one hydrogen bonding between amino acids inside the Wistar rat and human glucosidase active sites, indicating a strong binding.

The acute toxicity was tested using female rats; this study's findings suggest that *S. siamea* heartwood at a dose of 2000 mg/kg BW did not cause any treatment-related fatalities or clinical and histopathological toxicity. Therefore, systemic toxicity was not observed under the studied circumstances, and the LD_50_ was determined to be greater than 2000 mg/kg BW. Because diabetes mellitus is a chronic metabolic disease that requires long-term treatment, its safety data for long-term use are crucial. Therefore, subchronic toxicity of *S. siamea* heartwood extract at 250, 500, and 1000 mg/kg BW/day was evaluated in rats for 90 days. Body weight and food consumption provide a straightforward and sensitive tool to measure the toxic effects of chemicals and medications [40, 41].

Our findings from the subchronic toxicity showed no variations in body weight or food consumption between the treatment and control groups. These results revealed that the *S. siamea* heartwood extract had no effect on body weight or consumption of food. Nevertheless, there was a significant the end weight difference between the male and female rats. Male and female rats have different basal metabolisms and calorie expenditures, which might be the reason for the significant difference in final body weight. Furthermore, this experiment showed a slightly declining pattern in feed consumption, especially in male rats. According to another study, resveratrol treatment resulted in a drop in energy intake and an increase in metabolic rate. The regulating impact of resveratrol appears to be mediated by GLP-1, an incretin hormone mostly generated by intestinal endocrine cells. By slowing down the stomach's emptying process, bowel movements, and glycogen release, it can decrease the amount of food consumed [42].

The hematological parameters are important indices that can be employed to assess the toxicity of plant extracts in animal models [43]. From the results of this study, with the administration of *S. siamea* heartwood extract at doses of 500 and 1000 mg/kg BW, transient decreases were observed in hematocrit (Hct) and mean cell volume (MCV) values whereas transient increases were observed in MCHC and monocyte (mono) in female rats. A lack of synthesis of healthy red blood cells with proper size and shape may result in a substantial decrease in HCT. On the other hand, a low MCV is consistent with anemia and thalassemia syndromes [44]. Subchronic administration of *S. siamea* heartwood extract resulted in an increase in monocytes. According to this finding, the heartwood extract of *S. siamea* may include bioactive compounds, which enhance the immune system by enhancing the production of white blood cells [45].

BUN and Cr are used routinely to assess kidney function. These markers are commonly used to estimate the glomerular filtration rate [46]. BUN and Cr values of *S. siamea* heartwood extract 500 and 1000 groups were significantly increased in male rats as same as K^+^ and Ca^+^ levels. These results suggested that the doses of *S. siamea* heartwood extract at 500 and 1000 mg/kg BW possess toxicity to kidney tissue, which was consistent with the histopathological analysis of kidney.

Liver is an important organ which is involved in the biotransformation of drugs. Serum liver biomarker enzyme levels are biochemical indicators that are commonly assessed to evaluate the possibility of liver damage [47]. Histopathological studies serve as supportive evidence for biochemical analyses. The photomicrographs of sections of the liver of male rats treated orally with extracts of the *S. siamea* heartwood extract at a dose of 500 and 1000 mg/kg BW for 90 days showed histological changes more than female. The hydrolase ALP eliminates phosphate groups from a variety of compounds. ALP is a marker enzyme of the plasma membrane and endoplasmic reticulum, an enzyme present in the cells lining the bile duct of the gall bladder in the liver, and a byproduct of osteoblast activity [48]. Significant decreases in the levels of ALP were observed in both the male (250, 500, and 1000 mg/kg BW) and the female (1000 mg/kg BW) rats treated with the *S. siamea* heartwood extract in subchronic toxicity, as compared with the respective controls. The transaminases enzymes, SGOT and SGPT, are helpful biomarkers for potential toxicity. Numerous studies have established that while increased serum levels of hepatic transaminases (SGPT and SGOT) are not directly linked to liver injury, they are the cause of inflammation, cellular leakage, and damage to the cell membranes of the liver's cells [49]. The significant differences were observed in the levels of SGOT and SGPT in male rats at doses of 500 and 1000 mg/kg BW in this study, indicating that these doses of the *S. siamea* heartwood extract may cause side effects on the liver.

## 5. Conclusions

In conclusion, the results of this study showed that the *S. siamea* heartwood extract had significant inhibitory effects on *α*-glucosidase activity. In addition, the *S. siamea* heartwood extract could inhibit postprandial increases in blood glucose levels. The molecular docking of stilbenes, such as resveratrol, piceatannol, and dihydropiceatannol from this extract, was docked to *α*-glucosidase by using AutoDock Vina to predict the binding modes of these drug-like compounds. The results showed that resveratrol, piceatannol, and dihydropiceatannol possessed interactions with active site residues of the target protein, *α*-glucosidase. Lower dose of the *S. siamea* heartwood extract is safe to be administered for long-term use in prediabetes. This potential for drug discovery can also be a candidate for bioactivity targeting of *α*-glucosidase activity for used as a dietary supplement for diabetes. However, further studies will be conducted in the future on individuals who have chronic diabetes. This extract might be used in combination with other standard drugs. Consequently, determining the appropriate dosage of both standard drugs and extracts in vitro is crucial. In addition, diabetes must be induced in the animals (in vivo) before administering medications or extracts.

## Figures and Tables

**Table 1:** Clinical biochemistry values of control and rats treated with the *S. siamea* heartwood extract measured during the acute toxicity study.

Biochemical parameters	Control	2000 mg/kg BW
FBS	126.40 ± 2.23	116.60 ± 2.56⁣^∗^
BUN	21.25 ± 0.42	20.97 ± 0.91
Cr	0.56 ± 0.01	0.56 ± 0.02
Total protein	6.03 ± 0.24	5.74 ± 0.08
Albumin	3.40 ± 0.12	3.24 ± 0.09
Globulin	2.64 ± 0.14	2.50 ± 0.04
SGOT	109.80 ± 6.79	120.00 ± 12.24
SGPT	24.40 ± 1.75	20.20 ± 0.58
ALP	69.40 ± 3.91	69.40 ± 4.37
Total bilirubin	0.46 ± 0.01	0.46 ± 0.01
Direct bilirubin	0.11 ± 0.00	0.12 ± 0.00

Abbreviations: ALP = alkaline phosphatase, BUN = blood urea nitrogen, Cr = creatinine, FBS = fasting blood serum, SGOT = serum glutamic–oxaloacetic transaminase, SGPT = serum glutamate pyruvate transaminase.

⁣^∗^*p* < 0.05.

**Table 2:** Hematological values of control and rats treated with the *S. siamea* heartwood extract measured during the acute toxicity study.

Biochemical parameters	Control	2000 mg/kg BW
WBC	4.62 ± 0.68	4.62 ± 0.31
RBC	7.88 ± 0.18	7.90 ± 0.15
Hb	14.94 ± 0.25	14.82 ± 0.14
Hct	44.10 ± 0.89	44.02 ± 0.41
MCV	56.00 ± 0.50	55.80 ± 0.83
MCH	18.96 ± 0.15	18.78 ± 0.22
MCHC	33.86 ± 0.19	33.66 ± 0.15
Plt	765.40 ± 48.11	848.60 ± 38.96
RDW	14.86 ± 0.29	15.30 ± 0.43
PMN	6.72 ± 1.31	7.62 ± 0.98
Lymph	90.48 ± 1.37	89.12 ± 0.99
Mono	1.94 ± 0.36	2.20 ± 0.22
Eo	0.92 ± 0.22	1.06 ± 0.16
Baso	0.00 ± 0.00	0.00 ± 0.00

*Note:* Baso = basophils, Eo = eosinophils, Hb = hemoglobin, Hct = hematocrit, Lymph = lymphocytes, Mono = monocytes, Plt = platelets, RDW = red cell distribution width.

Abbreviations: MCH = mean corpuscular hemoglobin, MCHC = mean cell hemoglobin concentration, MCV = mean corpuscular volume, PMN = polymorphonuclear neutrophils, RBC = red blood cells, WBC = white blood cells.

**Table 3:** Clinical biochemistry values of male and female rats treated with the *S. siamea* heartwood extract measured during the subchronic toxicity study.

Biochemical parameters	Sex	Control	Dose of the *S. siamea* heartwood extract (mg/kg BW)		
			250	500	1000
FBS	Male	155.13 ± 4.95	148.88 ± 4.97	146.63 ± 4.08	144.88 ± 2.95
	Female	146.75 ± 3.74	144.50 ± 4.03	136.38 ± 6.63	121.50 ± 5.02

BUN	Male	18.27 ± 0.88	17.14 ± 0.65	26.41 ± 1.50⁣^∗∗^	25.14 ± 1.17⁣^∗∗^
	Female	22.09 ± 1.51	18.79 ± 0.77	19.84 ± 0.81	19.86 ± 1.00

Cr	Male	0.64 ± 0.01	0.57 ± 0.03	0.95 ± 0.03⁣^∗∗^	0.91 ± 0.02⁣^∗∗^
	Female	0.69 ± 0.04	0.75 ± 0.02	0.79 ± 0.02	0.75 ± 0.02

Cholesterol	Male	65.13 ± 3.31	65.25 ± 4.80	68.25 ± 3.71	70.75 ± 2.19
	Female	55.38 ± 3.21	51.75 ± 3.89	45.00 ± 5.17	42.88 ± 3.40

Total protein	Male	5.52 ± 0.13	5.82 ± 0.13	5.83 ± 0.09	6.40 ± 0.14
	Female	6.28 ± 0.18	6.03 ± 0.15	6.27 ± 0.22	6.19 ± 0.18

Albumin	Male	3.04 ± 0.03	3.05 ± 0.05	3.19 ± 0.03	3.33 ± 0.05
	Female	3.62 ± 0.06	3.47 ± 0.08	3.60 ± 0.09	3.59 ± 0.07

Globulin	Male	2.46 ± 0.12	2.77 ± 0.10	2.63 ± 0.10	3.07 ± 0.11
	Female	2.66 ± 0.19	2.56 ± 0.11	2.67 ± 0.14	2.60 ± 0.15

SGOT	Male	154.75 ± 4.84	125.50 ± 3.96	274.25 ± 21.40⁣^∗∗^	286.00 ± 12.68⁣^∗∗^
	Female	215.25 ± 12.43	244.38 ± 21.50	230.38 ± 11.67	258.63 ± 11.80

SGPT	Male	34.00 ± 1.60	33.25 ± 1.24	25.13 ± 1.42⁣^∗∗^	24.38 ± 2.02⁣^∗∗^
	Female	35.88 ± 2.02	48.13 ± 8.21	41.63 ± 3.95	46.00 ± 5.44

ALP	Male	79.13 ± 3.16	62.38 ± 2.27⁣^∗∗^	65.63 ± 3.52⁣^∗∗^	67.50 ± 1.67⁣^∗^
	Female	45.50 ± 1.25	43.63 ± 2.18	41.381.91	38.13 ± 0.64⁣^∗^

Total bilirubin	Male	0.49 ± 0.01	0.48 ± 0.01	0.42 ± 0.01	0.41 ± 0.01
	Female	0.41 ± 0.01	0.42 ± 0.01	0.45 ± 0.00	0.42 ± 0.01

Na^+^	Male	145.13 ± 0.35	145.13 ± 0.48	152.25 ± 0.49⁣^∗∗^	152.00 ± 0.89⁣^∗∗^
	Female	148.75 ± 0.45	149.00 ± 0.96	148.00 ± 0.50	146.50 ± 0.42

K^+^	Male	4.45 ± 0.07	4.41 ± 0.09	8.00 ± 0.32⁣^∗∗^	7.80 ± 0.28⁣^∗∗^
	Female	4.01 ± 0.14	3.88 ± 0.08	3.88 ± 0.10	3.89 ± 0.11

Abbreviations: ALP = alkaline phosphatase, BUN = blood urea nitrogen, Cr = creatinine, FBS = fasting blood serum, SGOT = serum glutamic–oxaloacetic transaminase, SGPT = serum glutamate pyruvate transaminase.

⁣^∗^*p* < 0.05.

⁣^∗∗^*p* ≤ 0.01.

**Table 4:** Hematological values of male and female rats treated with the *S. siamea* heartwood extract measured during the subchronic toxicity study.

Parameters	Sex	Control	Dose of the *S. siamea* heartwood extract (mg/kg BW)		
			250	500	1000
WBC (10^3^/μL)	Male	5.14 ± 0.54	4.88 ± 0.34	4.58 ± 0.58	5.60 ± 0.40
	Female	3.35 ± 0.32	3.25 ± 0.23	3.20 ± 0.33	3.25 ± 0.35

RBC (10^6^/μL)	Male	8.85 ± 0.14	8.86 ± 0.13	8.84 ± 0.14	8.80 ± 0.15
	Female	7.74 ± 0.07	7.94 ± 0.13	7.90 ± 0.10	7.87 ± 0.12

Hb (g/dL)	Male	15.38 ± 0.21	15.28 ± 0.10	15.30 ± 0.23	15.23 ± 0.22
	Female	14.43 ± 0.14	14.76 ± 0.23	14.66 ± 0.20	14.81 ± 0.51

Hct (%)	Male	44.00 ± 0.60	44.48 ± 0.46	43.26 ± 0.58	42.99 ± 0.68
	Female	45.60 ± 0.39	46.00 ± 0.70	44.58 ± 0.56	43.25 ± 0.43⁣^∗^

MCV (fL)	Male	49.73 ± 0.47	50.25 ± 0.89	48.94 ± 0.47	48.93 ± 0.65
	Female	58.96 ± 0.30	57.95 ± 0.32	56.45 ± 0.74⁣^∗^	55.04 ± 1.04⁣^∗∗^

MCH (pg)	Male	17.36 ± 0.11	17.25 ± 0.19	17.30 ± 0.18	17.31 ± 0.16
	Female	18.66 ± 0.16	18.60 ± 0.08	18.55 ± 0.15	18.84 ± 0.27

MCHC (g/dL)	Male	34.94 ± 0.19	34.35 ± 0.25	35.35 ± 0.13	35.44 ± 0.21
	Female	31.64 ± 0.13	32.06 ± 0.12	32.91 ± 0.30⁣^∗∗^	34.25 ± 0.29⁣^∗∗^

Plt (10^3^/μL)	Male	835.88 ± 26.70	850.88 ± 33.81	791.25 ± 103.27	839.13 ± 82.54
	Female	730.88 ± 34.75	696.75 ± 55.20	725.63 ± 53.56	613.15 ± 47.45

RDW	Male	20.01 ± 0.29	20.61 ± 0.28	20.10 ± 0.37	20.00 ± 0.23
	Female	17.68 ± 0.23	18.19 ± 0.28	17.26 ± 0.42	16.36 ± 0.34

PMN	Male	13.08 ± 1.79	14.20 ± 1.06	15.50 ± 0.97	20.61 ± 4.10
	Female	12.54 ± 1.44	16.78 ± 3.53	18.31 ± 2.47	21.05 ± 3.44

Lymp (%)	Male	81.58 ± 2.31	80.78 ± 1.15	79.66 ± 1.06	74.79 ± 4.73
	Female	84.30 ± 1.69	78.16 ± 3.96	76.83 ± 2.55	72.64 ± 3.49

Mono (%)	Male	2.19 ± 0.17	2.28 ± 0.12	2.63 ± 0.33	2.46 ± 0.39
	Female	1.66 ± 0.35	3.13 ± 0.68	3.64 ± 0.52⁣^∗^	3.80 ± 0.21⁣^∗^

Eo (%)	Male	3.16 ± 0.85	2.75 ± 0.55	2.11 ± 0.24	2.14 ± 0.47
	Female	1.50 ± 0.19	1.94 ± 0.42	1.23 ± 0.11	2.51 ± 0.74

Baso (%)	Male	0.00	0.00	0.00	0.00
	Female	0.00	0.00	0.00	0.00

*Note:* Baso = basophils, Eo = eosinophils, Hb = hemoglobin, Hct = hematocrit, Lymph = lymphocytes, Mono = monocytes, Plt = platelets, RDW = red cell distribution width.

Abbreviation: MCH = mean corpuscular hemoglobin, MCHC = mean cell hemoglobin concentration, MCV = mean corpuscular volume, PMN = polymorphonuclear neutrophils, RBC = red blood cells, WBC = white blood cells.

⁣^∗^*p* < 0.05.

⁣^∗∗^*p* ≤ 0.01.

**Table 5:** Scores of lesions in the liver for comparisons between the test group and control (subchronic toxicity) (*n* = 4).

No.	Control	Male (mg/kg BW)			Female (mg/kg BW)		
		250	500	1000	250	500	1000
1	0	0.25	2	3	1	2	2
2	0	0.75	2.5	3	1	2	2
3	0	1	2.5	4	1	2	2
4	0	1	7	2.25	1	2	2
Average	0	0.75⁣^∗^	2.50⁣^∗^	3.06⁣^∗^	1⁣^∗^	2⁣^∗^	2⁣^∗^

⁣^∗^*p* < 0.05.

**Table 6:** Scores of lesions in the kidney for comparisons between the test group and control (subchronic toxicity) (*n* = 4).

No.	Control	Male (mg/kg BW)			Female (mg/kg BW)		
		250	500	1000	250	500	1000
1	0	0	3	4.25	0	3	5.75
2	0	0	1.75	4.25	0.25	1.75	4.75
3	0	0	6.50	4.00	0.75	6.50	5
4	0	0	3.50	3.76	0.50	3.50	5.25
Average	0	0	3.69⁣^∗^	4.14⁣^∗^	0.38	4.31⁣^∗^	5.19⁣^∗^

⁣^∗^*p* < 0.05.

## Data Availability

The data that support the findings of this study are available in the supporting information of this article.

## Supporting Information

Figure S1: Docking validation result of human glucosidase (human sucrase–isomaltase, PDB ID: 3LPP). The RMSD value between the native ligand and the redocked ligand is 1.886 Å, lower than 3 Å (an acceptance criterion). The blue ligand is native, while the pink color ligand is redocked.

Figure S2: Docking validation result of Wistar rat glucosidase (Wistar rat sucrase–isomaltase, AFDB accession no: AF-P23739-F1). The RMSD value between the native ligand and the redocked ligand is 1.714 Å, lower than 3 Å (an acceptance criterion). The blue ligand is native, while the pink color ligand is redocked.

Table S1: Physical and behavior observation in rats during the acute toxicity study of the *S. siamea* heartwood extract.

Table S2: Physical and behavior observation in male and female rats during the subchronic toxicity study of the *S. siamea* heartwood extract.

## References

[1] Tanty H., Permai S. D., Pudjihastuti H. (2018). In Vivo Anti-Diabetic Activity Test of Ethanol Extract of the Leaves of Cassia Siamea Lamk. *Procedia Computer Science*.

[2] Uddin M. J., Ahamad M. M., Hoque M. N. (2023). A Comparison of Machine Learning Techniques for the Detection of Type-2 Diabetes Mellitus: Experiences From Bangladesh. *Information*.

[3] Van de Laar F. A., Lucassen P. L., Akkermans R. P., Van de Lisdonk E. H., Rutten G. E., Van Weel C. (2005). Alpha-Glucosidase Inhibitors for Type 2 Diabetes Mellitus. *Cochrane Database of Systematic Reviews*.

[4] Dirir A. M., Daou M., Yousef A. F., Yousef L. F. (2022). A Review of Alpha-Glucosidase Inhibitors From Plants as Potential Candidates for the Treatment of Type-2 Diabetes. *Phytochemistry Reviews*.

[5] Habtemariam S., Varghese G. K. (2017). Antioxidant, Anti-Alpha-Glucosidase and Pancreatic Beta-Cell Protective Effects of Methanolic Extract of Ensete Superbum Cheesm Seeds. *Asian Pacific Journal of Tropical Biomedicine*.

[6] Doan H. V., Riyajan S., Iyara R., Chudapongse N. (2018). Antidiabetic Activity, Glucose Uptake Stimulation and *α*-glucosidase Inhibitory Effect of Chrysophyllum Cainito L. Stem Bark Extract. *Bio Medical Central Complementary and Alternative Medicine*.

[7] Shailajan S., Gurjar D. (2014). Pharmacognostic and Phytochemical Evaluation of Chrysophyllum Cainito Linn. Leaves. *International Journal of Pharmaceutical Sciences Review and Research*.

[8] Ganesan S., Natesan S. K. (2017). Antidiabetic and Antihyperlipidaemic Activities of Ensete Superbum Fractions on High Fat Fed With Low Dose Streptozotocin Induced Type-2 Diabetes in Rats. *International Journal of Chemical and Pharmaceutical Analysis*.

[9] Goldson Barnaby A., Reid R., Warren D. (2016). Antioxidant Activity, Total Phenolics and Fatty Acid Profile of Delonix Regia, Cassia Fistula, *Spathodea campanulata*, *Senna Siamea* and Tibouchina Granulosa. *Journal of Analytical & Pharmaceutical Research*.

[10] Padumanonda T., Gritsanapan W. (2006). Barakol Contents in Fresh and Cooked Senna Siamea Leaves. *Southeast Asian Journal of Tropical Medicine and Public Health*.

[11] Balami V. M., Yakubu J., Mbaya H. A., Sodipo O. A., Khan I. Z. (2021). Nutritional Content, Phytochemical Evaluation and Antipyretic Effect of Methanol Leaf Extract of Senna Siamea (Kassod Tree). *Bulletin of Pure & Applied Sciences-Chemistry*.

[12] Smith Y. A. (2009). Determination of Chemical Composition of Senna-Siamea (Cassia Leaves). *Pakistan Journal of Nutrition*.

[13] Kamagaté M., Koffi C., Kouamé N. M. (2014). Ethnobotany, Phytochemistry, Pharmacology and Toxicology Profiles of Cassia Siamea Lam. *The Journal of Phytopharmacology*.

[14] Morita H., Oshimi S., Hirasawa Y. (2007). Cassiarins A and B, Novel Antiplasmodial Alkaloids From Cassia Siamea. *Organic Letters*.

[15] Ajaiyeoba E. O., Ashidi J. S., Okpako L. C., Houghton P. J., Wright C. W. (2008). Antiplasmodial Compounds From Cassia Siamea Stem Bark Extract. *Phytotherapy Research*.

[16] Nsonde Ntandou G. F., Banzouzi J. T., Mbatchi B. (2010). Analgesic and Anti-inflammatory Effects of Cassia Siamea Lam. Stem Bark Extracts. *Journal of Ethnopharmacology*.

[17] Majji L. N., Battu G. R., Jangiti R. K., Talluri M. R. (2013). Evaluation of In-Vitro Antibacterial Activity of Cassia Siamea Leaves. *International Journal of Pharmacy and Pharmaceutical Sciences*.

[18] Kumar S., Kumar V., Prakash O. M. (2010). Antidiabetic and Anti-lipemic Effects of Cassia Siamea Leaves Extract in Streptozotocin Induced Diabetic Rats. *Asian Pacific Journal of Tropical Medicine*.

[19] Nuankaew W., Heemman A., Wattanapiromsakul C. (2021). Anti-Insulin Resistance Effect of Constituents From Senna Siamea on Zebrafish Model, Its Molecular Docking, and Structure-Activity Relationships. *Journal of Natural Medicines*.

[20] Abdelsalam S. S., Korashy H. M., Zeidan A., Agouni A. (2019). The Role of Protein Tyrosine Phosphatase (PTP)-1B in Cardiovascular Disease and Its Interplay with Insulin Resistance. *Biomolecules*.

[21] Arulmozhiraja S., Matsuo N., Ishitsubo E., Okazaki S., Shimano H., Tokiwa H. (2016). Comparative Binding Analysis of Dipeptidyl Peptidase IV (DPP-4) With Antidiabetic Drugs-An Ab Initio Fragment Molecular Orbital Study. *PLoS One*.

[22] Dej-adisai S., Pitakbut T. (2015). Determination of A-Glucosidase Inhibitory Activity From Selected Fabaceae Plants. *Pakistan journal of pharmaceutical sciences*.

[23] Adisakwattana S., Yibchok-Anun S., Charoenlertkul P., Wongsasiripat N. (2011). Cyanidin-3-Rutinoside Alleviates Postprandial Hyperglycemia and Its Synergism With Acarbose by Inhibition of Intestinal *α*-Glucosidase. *Journal of Clinical Biochemistry & Nutrition*.

[24] Oecd (2022). Test No. 425: Acute Oral Toxicity: Up-and-Down Procedure. *OECD Guidelines for the Testing of Chemicals, Section 4*.

[25] Oecd (2018). Test No. 408: Repeated Dose 90-Day Oral Toxicity Study in Rodents. *OECD Guidelines for the Testing of Chemicals, Section 4*.

[26] Jumper J., Evans R., Pritzel A. (2021). Highly Accurate Protein Structure Prediction With AlphaFold. *Nature*.

[27] Varadi M., Anyango S., Deshpande M. (2022). Alpha Fold Protein Structure Database: Massively Expanding the Structural Coverage of Protein-Sequence Space With High-Accuracy Models. *Nucleic Acids Research*.

[28] Pettersen E. F., Goddard T. D., Huang C. C. (2004). UCSF Chimera-A Visualization System for Exploratory Research and Analysis. *Journal of Computational Chemistry*.

[29] Eberhardt J., Santos-Martins D., Tillack A. F., Forli S. (2021). AutoDock Vina 1.2.0: New Docking Methods, Expanded Force Field, and Python Bindings. *Journal of Chemical Information and Modeling*.

[30] Dej-adisai S., Rais I. R., Wattanapiromsakul C., Pitakbut T. (2021). Phytochemical Investigation of Bauhinia Winitii Based on Alpha-Glucosidase Inhibitory Effect and Molecular Docking Affirmation. *Pharmacognosy Magazine*.

[31] Laskowski R. A., Swindells M. B. (2011). LigPlot+: Multiple Ligand-Protein Interaction Diagrams for Drug Discovery. *Journal of Chemical Information and Modeling*.

[32] Sim L., Willemsma C., Mohan S., Naim H. Y., Pinto B. M., Rose D. R. (2010). Structural Basis for Substrate Selectivity in Human Maltase-Glucoamylase and Sucrase-Isomaltase N-Terminal Domains. *Journal of Biological Chemistry*.

[33] Lautié E., Russo O., Ducrot P., Boutin J. A. (2020). Unraveling Plant Natural Chemical Diversity for Drug Discovery Purposes. *Frontiers in Pharmacology*.

[34] Ismail A. H., Idris A. N., Amina M. M., Ibrahim A. S., Audu S. A. (2015). Phytochemical Studies and Thin Layer Chromatography of Leaves and Flower Extracts of Senna Siamea Lam for Possible Biomedical Applications. *Journal of Pharmacognosy and Phytotherapy*.

[35] Giri B., Dey S., Das T., Sarkar M., Banerjee J., Dash S. K. (2018). Chronic Hyperglycemia Mediated Physiological Alteration and Metabolic Distortion Leads to Organ Dysfunction, Infection, Cancer Progression and Other Pathophysiological Consequences: An Update on Glucose Toxicity. *Biomedicine & Pharmacotherapy*.

[36] Bischoff H. (1995). The Mechanism of Alpha-Glucosidase Inhibition in the Management of Diabetes. *Clinical and Investigative Medicine*.

[37] Miranda J., Eseberri I., Lasa A., Portillo M. P. (2018). Lipid Metabolism in Adipose Tissue and Liver From Diet-Induced Obese Rats: A Comparison Between Wistar and Sprague-Dawley Strains. *Journal of Physiology & Biochemistry*.

[38] Odason E. E., Kolawole J. A. (2007). Effect of the Aqueous Extract of the Root of Cassia Siamea Lam. (Ceasalpiniaceae). *Nigerian Journal of Pharmaceutical Research*.

[39] Toeller M. (1994). *α*-Glucosidase Inhibitors in Diabetes: Efficacy in NIDDM Subjects. *European Journal of Clinical Investigation*.

[40] Olayode O. A., Daniyan M., Olayiwola G. (2020). Biochemical, Hematological and Histopathological Evaluation of the Toxicity Potential of the Leaf Extract of Stachytarpheta Cayennensis in Rats. *Journal of Traditional and Complementary Medicine*.

[41] Bilan V. P., Salah E. M., Bastacky S. (2011). Diabetic Nephropathy and Long-Term Treatment Effects of Rosiglitazone and Enalapril in Obese ZSF1 Rats. *Journal of Endocrinology*.

[42] Dao T. M., Waget A., Klopp P. (2011). Resveratrol Increases Glucose Induced GLP-1 Secretion in Mice: A Mechanism Which Contributes to the Glycemic Control. *PLoS One*.

[43] Sunmonu T. O., Oloyede O. B. (2010). Performance and Haematological Indices in Rats Exposed to Monocrotophos Contamination. *Human & Experimental Toxicology*.

[44] Arika W. M., Nyamai D. W. (2016). Hematological Markers of In Vivo Toxicity. *Journal of Hematology and Thromboembolic Diseases*.

[45] Benrahou K., Mrabti H. N., Assaggaf H. M. (2022). Acute and Subacute Toxicity Studies of Erodium Guttatum Extracts by Oral Administration in Rodents. *Toxins*.

[46] Griffin B. R., Faubel S., Edelstein C. L. (2019). Biomarkers of Drug-Induced Kidney Toxicity. *Therapeutic Drug Monitoring*.

[47] Vaja R., Rana M. (2020). Drugs and the Liver. *Anaesthesia and Intensive Care Medicine*.

[48] Shahjahan M., Sabitha K., Jainu M., Shyamala Devi C. S. (2004). Effect of Solanum Trilobatum Against Carbon Tetrachloride Induced Hepatic Damage in Albino Rats. *Indian Journal of Medical Research*.

[49] Herlina T., Madihah M., Deni D., Amien S. (2017). Subchronic Toxicity of Methanol Extract From Erythrina Variegata (Leguminosae) Leaves on Male Wistar Rats (*Rattus norvegicus*). *Molekul*.

