# Supplementary material for: Antidiabetic Potential of Senna siamea: α-Glucosidase Inhibition, Postprandial Blood Glucose Reduction, Toxicity Evaluation, and Molecular Docking
Source: Scientifica (Cairo). 2025 Jan 23;2025:6650349. doi: 10.1155/sci5/6650349 (PMC11824848; doi:10.1155/sci5/6650349)
Supplement: Supporting Information — Additional supporting information can be found online in the Supporting Information section. [file 6650349.f1.docx]

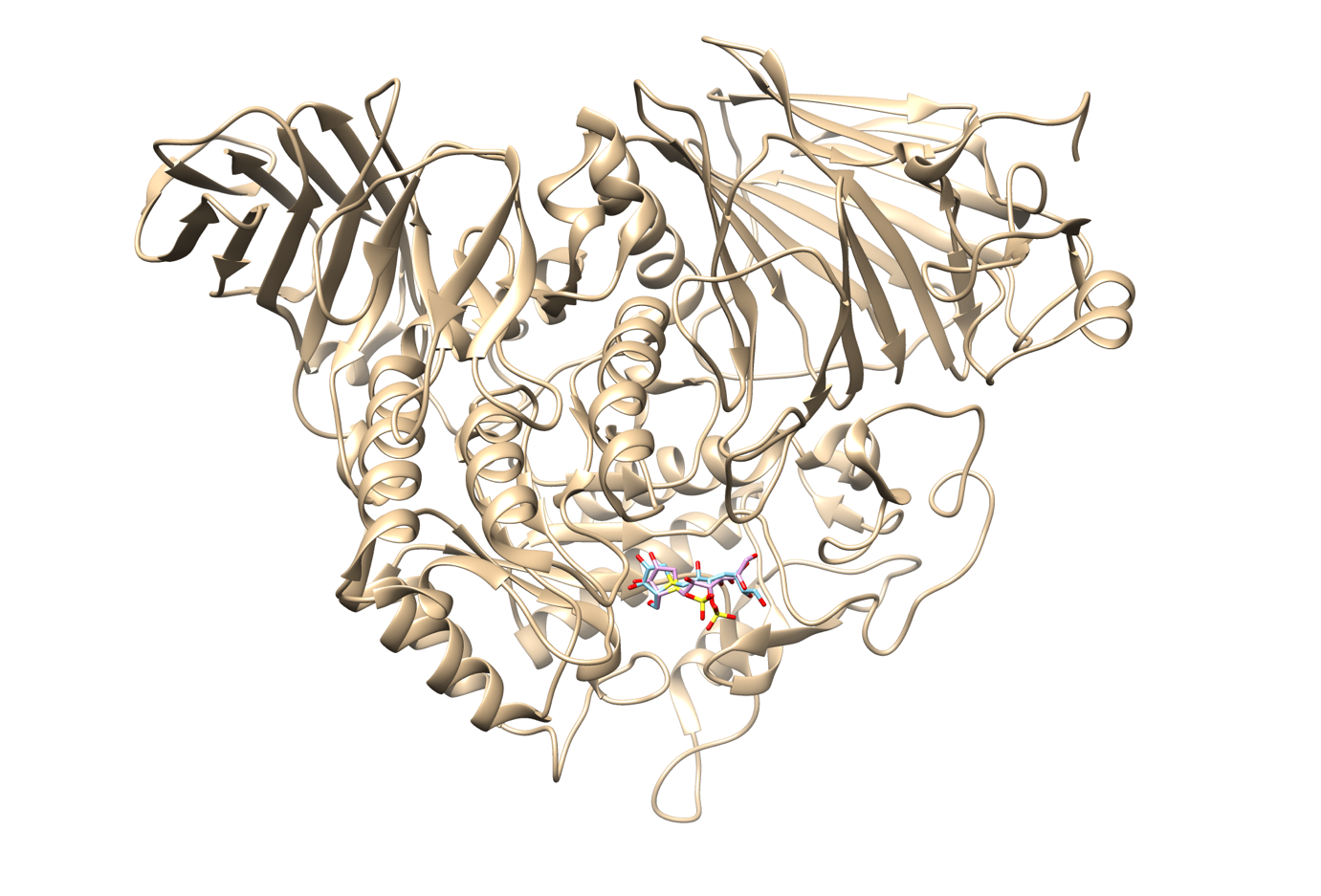


**Figure S1**. Docking validation result of human glucosidase (human sucrase–isomaltase, PDB ID: 3LPP). The RMSD value between the native ligand and the re-docked ligand is 1.886 Å, lower than 3 Å (an acceptance criterion). The blue ligand is native, while the pink color ligand is re-docked.


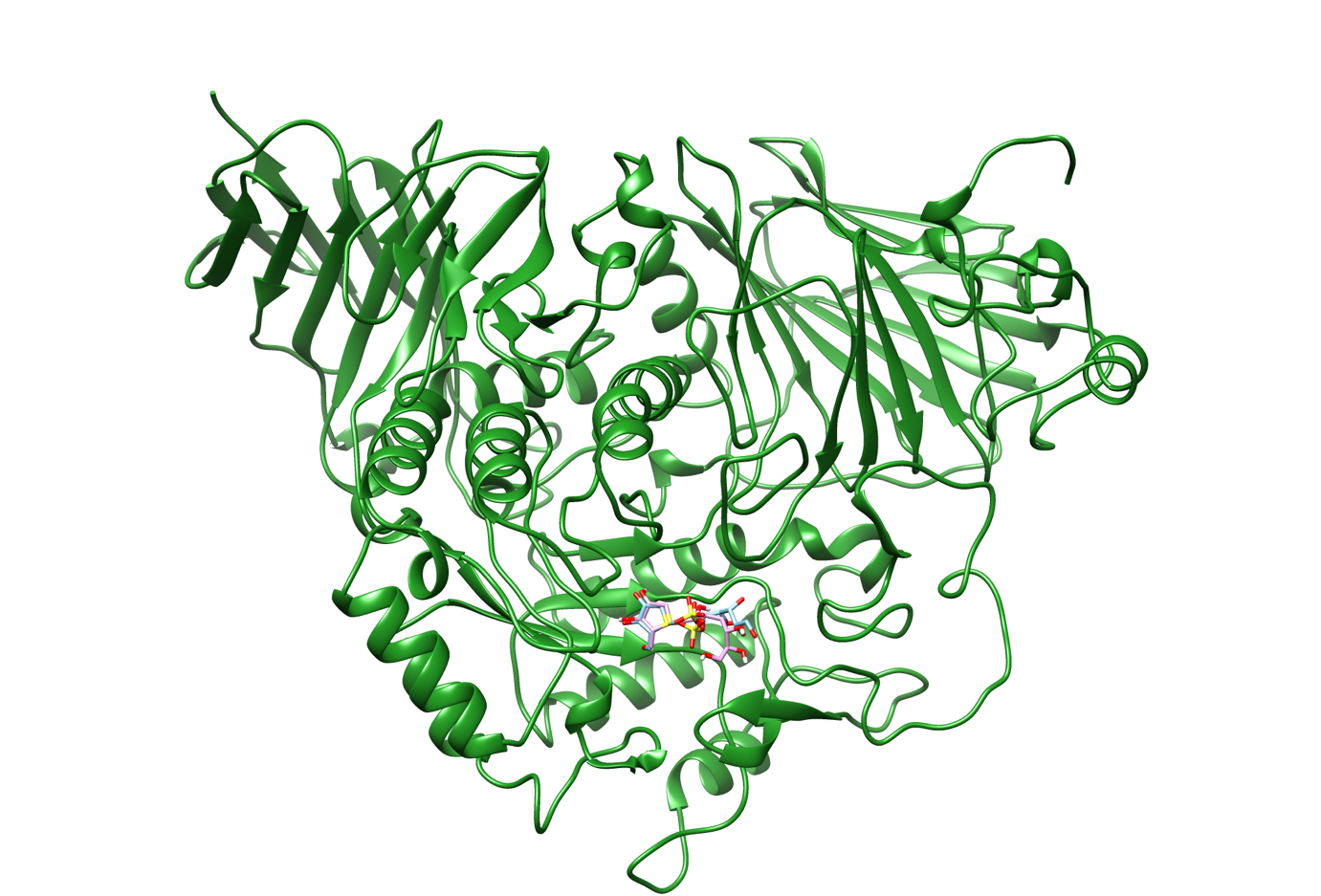


**Figure S2.** Docking validation result of Wistar rat glucosidase (Wistar rat sucrase–isomaltase, AFDB accession no: AF-P23739-F1). The RMSD value between the native ligand and the re-docked ligand is 1.714 Å, lower than 3 Å (an acceptance criterion). The blue ligand is native, while the pink color ligand is re-docked.

**Table S1** Physical and behavior observation in rats during the acute toxicity study of *S. siamea* heartwood extract

| Symptoms | Symptom determination in rats at different time | | | | | | | | | | | | | | | | | | | | | |
| --- | --- | --- | --- | --- | --- | --- | --- | --- | --- | --- | --- | --- | --- | --- | --- | --- | --- | --- | --- | --- | --- | --- |
|  | 30 min | | 1 h | | 2 h | | 3 h | | | 4 h | | 24 h | | | 48 h | | | 7 d | | | 14 d | |
|  | C | T | C | T | C | T | C | T | C | | T | C | T | C | | T | C | | T | C | | T |
| Fur & skin | N | N | N | N | N | N | N | N | N | | N | N | N | N | | N | N | | N | N | | N |
| Eyes | N | N | N | N | N | N | N | N | N | | N | N | N | N | | N | N | | N | N | | N |
| Salivation | N | N | N | N | N | N | N | N | N | | N | N | N | N | | N | N | | N | N | | N |
| Respiration | N | N | N | N | N | N | N | N | N | | N | N | N | N | | N | N | | N | N | | N |
| Urination(color) | N | N | N | N | N | N | N | N | N | | N | N | N | N | | N | N | | N | N | | N |
| Diarrhea/Feces | N | N | N | N | N | N | N | N | N | | N | N | N | N | | N | N | | N | N | | N |
| Somatomotor activity & behavior pattern | N | N | N | N | N | N | N | N | N | | N | N | N | N | | N | N | | N | N | | N |
| Sleep/Lethargy | N | N | N | N | N | N | N | N | N | | N | N | N | N | | N | N | | N | N | | N |
| Mucous membrane | N | N | N | N | N | N | N | N | N | | N | N | N | N | | N | N | | N | N | | N |
| Convulsions & tremors | N | N | N | N | N | N | N | N | N | | N | N | N | N | | N | N | | N | N | | N |
| Itching | N | N | N | N | N | N | N | N | N | | N | N | N | N | | N | N | | N | N | | N |
| Coma | - | - | - | - | - | - | - | - | - | | - | - | - | - | | - | - | | - | - | | - |
| Mortality | - | - | - | - | - | - | - | - | - | | - | - | - | - | | - | - | | - | - | | - |

Note: N = normal, - = no sign, C = control group, T = treatment group with *S. siamea* heartwood extract at 2,000 mg/kg BW.

**Table S2** Physical and behavior observation in male and female rats during the sub-chronic toxicity study of *S. siamea* heartwood extract

| Symptoms | Symptom determination in rats at different time | | | | | | | | | | | | | | | | | | | | | | | | | |
| --- | --- | --- | --- | --- | --- | --- | --- | --- | --- | --- | --- | --- | --- | --- | --- | --- | --- | --- | --- | --- | --- | --- | --- | --- | --- | --- |
|  | 30 min | | | | 24 h | | | | 14 d | | | | 28 d | | | | | 60 d | | | | | 90 d | | | |
|  | 1 | 2 | 3 | 4 | 1 | 2 | 3 | 4 | 1 | 2 | 3 | 4 | 1 | 2 | 3 | 4 | 1 | | 2 | 3 | 4 | 1 | | 2 | 3 | 4 |
| Fur & skin | N | N | N | N | N | N | N | N | N | N | N | N | N | N | N | N | N | | N | N | N | N | | N | N | N |
| Eyes | N | N | N | N | N | N | N | N | N | N | N | N | N | N | N | N | N | | N | N | N | N | | N | N | N |
| Salivation | N | N | N | N | N | N | N | N | N | N | N | N | N | N | N | N | N | | N | N | N | N | | N | N | N |
| Respiration | N | N | N | N | N | N | N | N | N | N | N | N | N | N | N | N | N | | N | N | N | N | | N | N | N |
| Urination(color) | N | N | N | N | N | N | N | N | N | N | N | N | N | N | N | N | N | | N | N | N | N | | N | N | N |
| Diarrhea | N | N | N | N | N | N | N | N | N | N | N | N | N | N | N | N | N | | N | N | N | N | | N | N | N |
| Somatomotor activity & behavior pattern | N | N | N | N | N | N | N | N | N | N | N | N | N | N | N | N | N | | N | N | N | N | | N | N | N |
| Sleep/Lethargy | N | N | N | N | N | N | N | N | N | N | N | N | N | N | N | N | N | | N | N | N | N | | N | N | N |
| Mucous membrane | N | N | N | N | N | N | N | N | N | N | N | N | N | N | N | N | N | | N | N | N | N | | N | N | N |
| Convulsions & tremors | N | N | N | N | N | N | N | N | N | N | N | N | N | N | N | N | N | | N | N | N | N | | N | N | N |
| Itching | N | N | N | N | N | N | N | N | N | N | N | N | N | N | N | N | N | | N | N | N | N | | N | N | N |
| Coma | - | - | - | - | - | - | - | - | - | - | - | - | - | - | - | - | - | | - | - | - | - | | - | - | - |
| Mortality | - | - | - | - | - | - | - | - | - | - | - | - | - | - | - | - | - | | - | - | - | - | | - | - | - |

Note: N = normal, P = present sign, - = no sign, 1= control group, 2 = 250 mg/kg of extract, 3 = 500 mg/kg of extract, 4 = 1000 mg/kg of extract
